# *Osmanthus fragrans* Flavonoid Extract Inhibits Adipogenesis and Induces Beiging in 3T3-L1 Adipocytes

**DOI:** 10.3390/foods13121894

**Published:** 2024-06-16

**Authors:** Zhiying Yang, Yuxin Lu, Tingting Li, Xunyong Zhou, Jia Yang, Shuwen Yang, Su Bu, Yifan Duan

**Affiliations:** 1 College of Life Science, Nanjing Forestry University, Nanjing 210037, China; zhiying@njfu.edu.cn (Z.Y.); yuxinlu@njfu.edu.cn (Y.L.); yangjia95@icloud.com (J.Y.); yangshuwen1120@163.com (S.Y.); yifanduan@njfu.edu.cn (Y.D.); 2 Department of Food Science and Technology, College of Light Industry and Food Engineer, Nanjing Forestry University, Nanjing 210037, China; litingting@njfu.edu.cn; 3 HC Enzyme (Shenzhen) Biotech. Co., Ltd., Shenzhen 518112, China; zimmernchow@126.com; 4 International Cultivar Registration Center for Osmanthus, Nanjing 210037, China

**Keywords:** *Osmanthus fragrans* flavonoid extract, adipogenesis, reactive oxygen species, mitochondrial biogenesis, beiging/browning, AMPK signaling pathway

## Abstract

*Osmanthus fragrans* has a long history of cultivation in Asia and is widely used in food production for its unique aroma, which has important cultural and economic values. It is rich in flavonoids with diverse pharmacological properties, such as antioxidant, anti-tumor, and anti-lipid activities. However, little is known regarding the effects of *Osmanthus fragrans* flavonoid extract (OFFE) on adipogenesis and pre-adipocyte transdifferentiation. Herein, this research aimed to investigate the effect of OFFE on the differentiation, adipogenesis, and beiging of 3T3-L1 adipocytes and to elucidate the underlying mechanism. Results showed that OFFE inhibited adipogenesis, reduced intracellular reactive oxygen species levels in mature adipocytes, and promoted mitochondrial biogenesis as well as beiging/browning in 3T3-L1 adipocytes. This effect was accompanied by increased mRNA and protein levels of the brown adipose-specific marker gene *Pgc-1a*, and the upregulation of the expression of *UCP1*, *Cox7A1*, and *Cox8B*. Moreover, the research observed a dose-dependent reduction in the mRNA expression of adipogenic genes (*C/EBPα*, *GLUT-4*, *SREBP-1C*, and *FASN*) with increasing concentrations of OFFE. Additionally, OFFE activated the AMPK signaling pathway to inhibit adipogenesis. These findings elucidate that OFFE has an inhibitory effect on adipogenesis and promotes browning in 3T3-L1 adipocytes, which lays the foundation for further investigation of the lipid-lowering mechanism of OFFE in vivo in the future.

## 1. Introduction

In 2025, one billion adults (approximately 12.5% of the world’s population) are expected to be obese, making obesity a serious challenge to public health systems around the world [1]. The rate of overweight and obesity among adults in China is as high as 65.3% [2].Obesity increases the risk of developing numerous diseases, such as metabolic disorders, type 2 diabetes, steatohepatitis, gallstones and COVID-19 [3]. Due to its rapidly increasing prevalence, obesity has evolved from being solely a health issue to a complex social, medical, and economic challenge [4]. The currently available interventions against obesity, such as caloric restriction, dietary modifications, lifestyle management, pharmacotherapy, gut flora modulation, and bariatric surgery, have demonstrated limited efficacy [5,6]. Consequently, there is a growing interest in exploring safe, effective, and ingestible natural bioactive ingredients as potential remedies [7,8,9].

Adipose tissue serves as a vital energy reservoir and endocrine organ in both humans and animals. It is essential for homeostatic energy regulation [10]. In mammals, adipose tissues have two types based on function: white adipose tissue (WAT) and brown adipose tissue (BAT). WAT primarily functions as an energy storage site and releases hormones that regulate systemic metabolism and insulin resistance [11,12]. In contrast, BAT is capable of dissipating energy and generating heat through the brown-fat-specific uncoupling protein 1 (UCP1) in the mitochondria. Targeting BAT-mediated thermogenesis may represent a feasible alternative approach to increase energy expenditure [13,14].

Within WAT, the third type of adipocytes, beige adipocytes exhibit similar morphology to brown adipocytes, with many mitochondria and multi-locular lipid droplets, though their properties overlap with white adipocytes [15]. Under specific stimuli, such as cold stress, a process called “browning” can cause white adipocytes to transform into beige adipocytes. This transformation exerts a potent thermogenic effect facilitated by UCP1, leading to increased energy expenditure owing to increased mitochondrial oxygen consumption [15]. Therefore, the induction of white adipocyte browning offers a viable approach to reduce fat storage by improving mitochondrial metabolic efficiency. The mitochondrial electron transport chain serves as the primary site for the production of reactive oxygen species (ROS) in adipocytes [16]. Excessive ROS production reduces mitochondrial biogenesis, whereas decreased ROS levels promote mitochondrial biogenesis, thus facilitating white adipocyte browning.

Adipogenesis is a complex process controlled by multiple transcription factors and enzymes that are also essential for lipid production and metabolism during the differentiation of 3T3-L1 preadipocytes. This intricate series of events includes synchronized alterations in hormone sensitivity and gene expression, where factors such as CCAAT/enhancer-binding protein-α (C/EBPα) [17], sterol regulatory element-binding protein-1c (SREBP-1C) [18], fatty acid synthase (FASN), and glucose transporter protein 4 (GLUT-4) play essential roles [19]. Among these, C/EBPα stands out as a pivotal transcription factor orchestrating the final stages of pre-adipocyte differentiation [20]. Adipocytes release hormonal signals that activate C/EBPα expression, which, in turn, regulates other genes to ultimately drive adipocyte differentiation [21]. SREBP-1C is primarily regulated at the transcriptional level and is highly expressed in adipose tissues, regulating FASN expression and involvement in adipocyte differentiation [22]. FASN and GLUT-4 are indispensable for adipocyte differentiation.

*Osmanthus fragrans*, an evergreen woody fragrant plant of *Osmanthus* Lour. in Oleaceae, is also known as sweet osmanthus and widely cultivated in East Asia. *O. fragrans* possesses valuable edible, medicinal, and aesthetic properties. It contains numerous bioactive components, such as flavonoids, polysaccharides, and polyphenolic compounds. These nutrients exhibit hypolipidemic, anti-diabetic, antiaging, antioxidant, anti-cancer, anti-inflammatory, hepatoprotective activity and immunostimulatory properties [23,24,25]. *O. fragrans* is widely used in the cosmetic industry [26,27]. *Osmanthus* essential oil is one of the ingredients in many high-end perfumes in Europe [28]. Cosmetic products with *O. fragrans* extract have whitening and brightening effects, increased resistance to oxidative damage, and inhibition of melanin formation and tyrosinase activity [27,29].

In addition, the floral aroma of *O. fragrans* has been found to directly inhibit appetite neuropeptides in the hypothalamus, which can contribute to reducing food intake and ultimately aiding in weight management [30]. It was shown that intragastric administration of cinnamon extract to mice for 15 consecutive days effectively inhibited acetaminophen-induced elevation of glutamine aminotransferase, ameliorated lipid peroxidation in the liver and lungs, and significantly reduced vacuolization in liver tissue [31]. Loganic acid, a major secondary metabolite of *O. fragrans*, reduces the expression of key lipogenesis-related genes, such as lipocalin and lipoprotein lipase, and has anti-adipogenic and anti-osteoporotic effects [32,33].

These findings suggest that the floral aroma and its active constituents hold substantial promise for mitigating metabolic disorders. However, limited research has addressed the regulatory effect of *O. fragrans* flavonoid extract (OFFE) on adipogenesis and white adipocyte browning. Therefore, this research aimed to investigate whether OFFE could trigger adipocyte browning and inhibiting adipogenesis in 3T3-L1 adipocytes as well as the underlying mechanism.

## 2. Results

### 2.1. OFFE’s Impact on 3T3-L1 Cellular Viability

**Figure 1:**
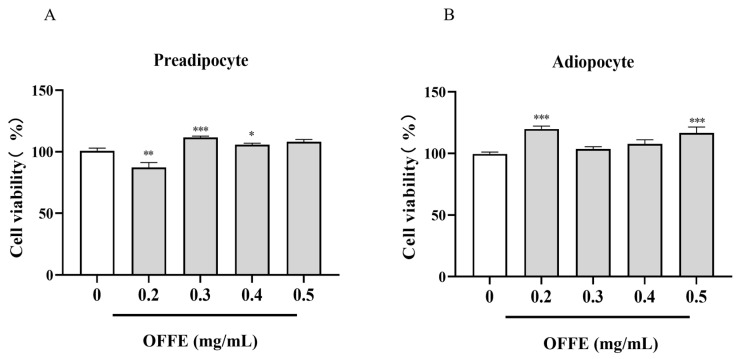
Effect of OFFE on the viability of 3T3-L1 cells. (**A**) pre-adipocytes and (**B**) adipocytes after 48 h of treatment. The data represent the mean ± standard error of the mean (SEM) from three independent experiments. * *p* < 0.05, ** *p* < 0.01, *** *p* < 0.001, compared with the control group.

MTT assays were performed to assess the effect of 48-h OFFE treatment on the viability of 3T3-L1. In pre-adipocytes, cell viability decreased by 25% following 0.2 mg/mL OFFE treatment (Figure 1A, *p* < 0.01). Other OFFE concentrations had almost no effect on viability. However, 0.3 mg/mL OFFE had a slight, yet significant effect on viability. In mature adipocytes, most of the tested concentrations had no effect on cell viability, except for 0.2 and 0.5 mg/mL, which significantly increased cell viability by 20% and 17% (Figure 1B, *p* < 0.001), respectively. Overall, OFFE exposure did not alter cell viability in both pre-adipocyte and adipocyte, albeit a modest decline was observed at specific doses.

### 2.2. OFFE Inhibits 3T3-L1 Adipogenesis

**Figure 2:**
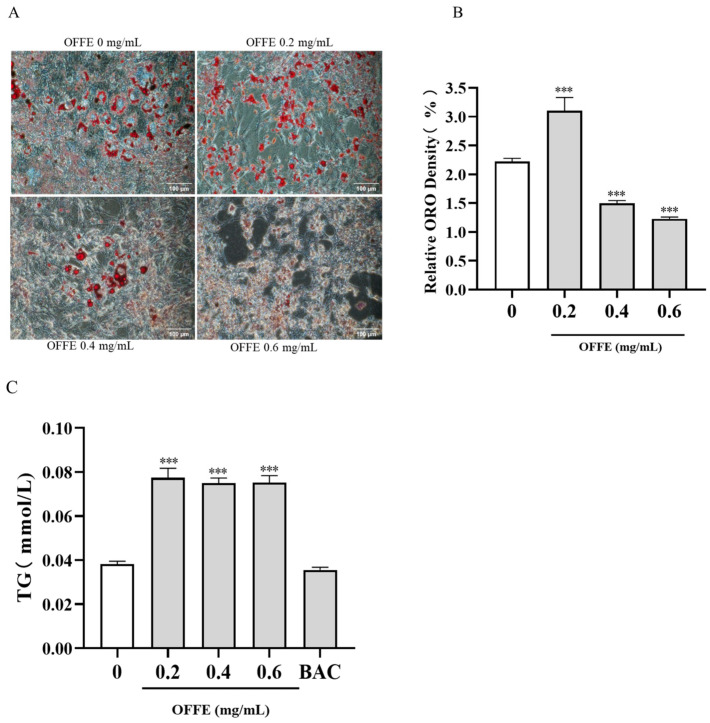
OFFE inhibited lipid accumulation in 3T3-L1 adipocytes and increased triglyceride secretion in vitro. (**A**) Representative images of ORO staining were taken at 200× (scale bar = 100 μm). (**B**) and semi-quantitative analysis was performed by dissolving ORO-stained lipid droplets in isopropanol. (**C**) The effect of OFFE treatment on extracellular TG content in 3T3-L1 mature adipocytes. Data are presented as the mean ± SEM from three independent experiments. *** *p* < 0.001, compared with the control group.

To evaluate how OFFE affects cell differentiation and lipid accumulation, ORO staining was conducted in mature adipocytes treated with or without OFFE during differentiation (days 3–8); the lipid droplets were photographed using reverse microscopy. This research observed lipid droplet accumulation in cells exposed to 0.2 mg/mL OFFE was markedly enhanced. However, 0.4 and 0.6 mg/mL OFFE-treated cells exhibited decreasing trends in cell proliferation and differentiation, reflecting a lower efficiency of lipid synthesis (Figure 2A). Isopropanol was used to dissolve the ORO dye, and the absorbance at 510 nm was semi-quantitatively analyzed. Compared with the control treatment, 0.2 mg/mL OFFE significantly promoted lipid accumulation, with a 39% increase. However, 0.4 and 0.6 mg/mL of OFFE induced significant inhibition of lipid accumulation, with a 33% and 45% decrease, respectively (Figure 2B, *p* < 0.001). This indicated that OFFE promotes lipid accumulation at lower concentrations while significantly inhibiting differentiation and lipid accumulation at higher concentrations.

Levels of extracellular triglycerides (TG) in the culture medium of mature adipocytes treated with OFFE were measured. Extracellular TG levels were significantly elevated in all groups. Treatment with 0.2, 0.4, and 0.6 mg/mL of OFFE significantly increased TG content by 103%, 96%, and 97%, respectively (Figure 2C, *p* < 0.001), relative to that in the control group. However, extracellular TG content in the BAC group decreased by 7% (Figure 2C, *p* < 0.001). This observation indicated that OFFE treatment prompted the release of triglycerides into the extracellular space, thereby reducing intracellular triglyceride levels and mitigating lipid accumulation.

### 2.3. OFFE Inhibits the Expression of Adipogenic Genes

**Figure 3:**
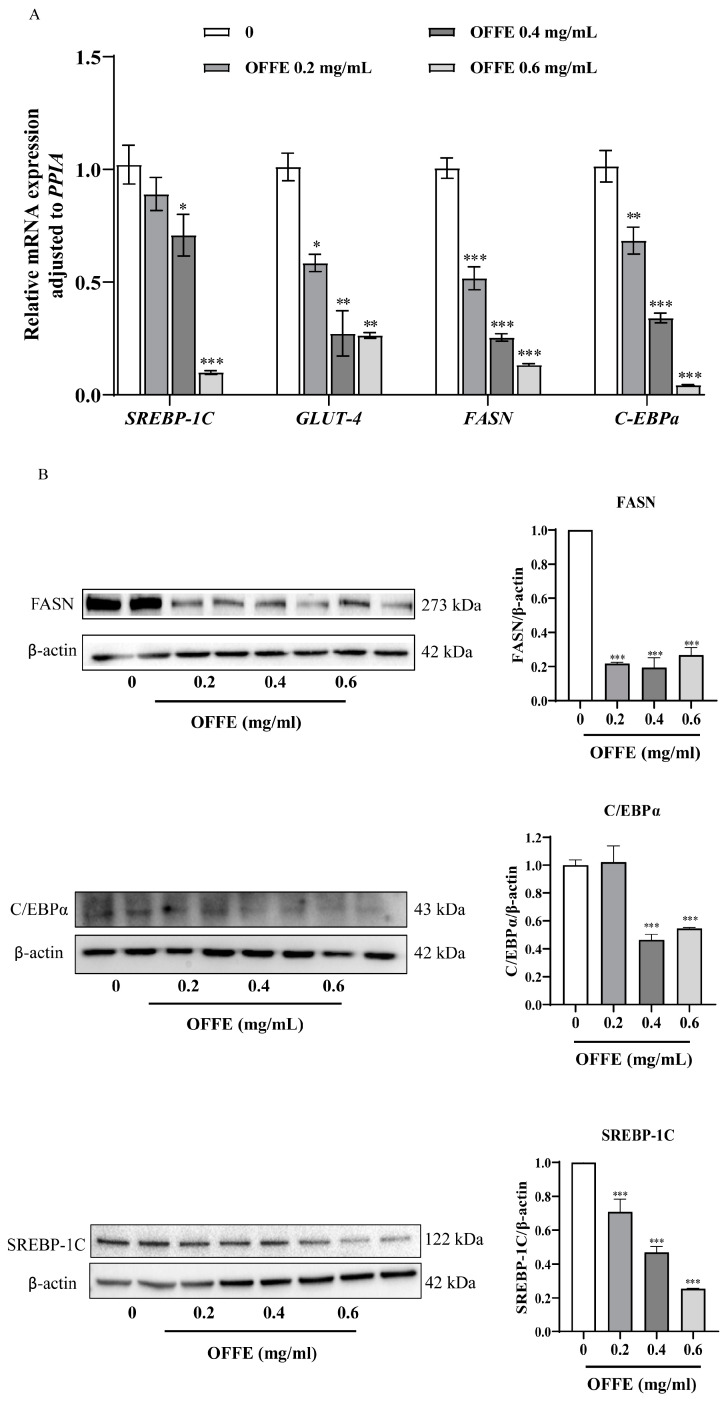
Effect of OFFE on the mRNA and protein expression of adipogenic genes. (**A**) OFFE treatment downregulated the relative mRNA expression of *SREBP-1C*, *GLUT-4*, *FASN*, and *C/EBPα*. (**B**) Western blot was used to detect the protein levels of SREBP-1C, FASN, and CEBPα. β-Actin was used as the loading control. The intensity of each band was quantified using densitometry analysis. The values are expressed as the mean ± SEM of three independent experiments. Each value was normalized to a control, and the control was set to 100%. * *p* < 0.05, ** *p* < 0.01, *** *p* < 0.001, compared with the control group.

SREBP-1c and C/EBPα are central transcription factors in adipogenesis. Their relative mRNA expression was examined in mature adipocytes after OFFE treatment from days 3 to 8 during the differentiation process. As shown in Figure 3A, higher OFFE concentrations resulted in a decline in the mRNA levels of *SREBP-1C* and *C/EBPα*. Specifically, treatment with 0.2 mg/mL reduced *SREBP-1C* and *C/EBPα* mRNA levels by 13% and 33% (*p* < 0.01), respectively. Treatment with 0.4 mg/mL resulted in a downregulation of mRNA expression by 31.3% (*p* < 0.05) and 67.3% (*p* < 0.001), respectively. However, treatment with 0.6 mg/mL of OFFE reduced these levels by 92% (*p* < 0.001) and 97% (*p* < 0.001), respectively.

The relative mRNA expression of two adipogenic genes, *GLUT-4* and *FASN*, was also measured. A significant OFFE concentration-dependent decrease was noted for both *GLUT-4* and *FASN*. Concentrations of 0.2, 0.4, and 0.6 mg/mL downregulated *GLUT-4* expression by 42.6% (*p* < 0.05), 73.9% (*p* < 0.01), and 74.7% (*p* < 0.01), respectively. Similarly, *FASN* mRNA expression decreased by 48.9%, 75.2%, and 87.3%, respectively (Figure 3A, *p* < 0.001). These results demonstrated that OFFE has a significant dose-dependent inhibitory effect on *GLUT-4* and *FASN* expression. Aligned with the qRT-PCR results, the protein levels of *FASN*, *C/EBPα*, and *SREBP-1C* showed a dose-dependent decrease after OFFE treatment (Figure 3B, *p* < 0.001). Taken together, OFFE significantly suppressed the expression of adipogenic genes to inhibit adipogenesis in 3T3-L1 adipocytes.

### 2.4. OFFE Regulates ROS Content in 3T3-L1 Mature Adipocytes

The mitochondrial electron transport chain is the major source and target of ROS [34]. Cellular redox homeostasis pivots on ROS dynamics. Importantly, excess ROS accumulation may impair mitochondrial function [35].

**Figure 4:**
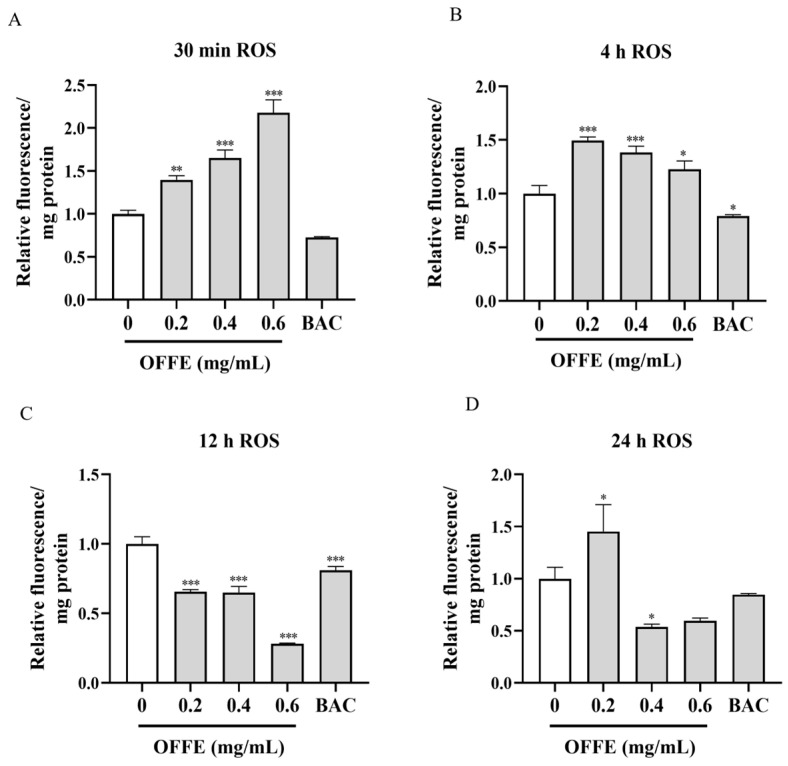
Effects of different OFFE concentrations on ROS levels in mature 3T3-L1 adipocytes at different time points. ROS fluorescence intensity in 3T3-L1 adipocytes treated with OFFE for 30 min (**A**), 4 h (**B**), 12 h (**C**), and 24 h (**D**). Data are presented as the mean ± SEM of three independent experiments. * *p* < 0.05, ** *p* < 0.01, *** *p* < 0.001, compared with the control group.

The 3T3-L1 mature adipocytes were exposed to OFFE, and ROS assays were conducted at various time points (30 min, 4, 12, and 24 h) (Figure 4). After 30 min of OFFE treatment, ROS levels increased in a dose-dependent manner. The relative ROS fluorescence intensity (normalized to mg protein) increased by 40% (*p* < 0.01) and up to 118%, respectively (*p* < 0.001), upon treatment with 0.2 and 0.6 mg/mL of OFFE (Figure 4A). Conversely, the BAC group did not exhibit an elevation in ROS levels when compared to the control group. At 4 h post-treatment, although the relative ROS fluorescence intensity was significantly elevated in all OFFE-treated groups relative to that in the control group, a decreasing trend was noted with increasing OFFE concentrations. The relative fluorescence intensity of ROS in the 0.2 mg/mL OFFE-treated group increased significantly by 49% (Figure 4B, *p* < 0.001), whereas that in the 0.6 mg/mL OFFE-treated group increased by 23% (Figure 4B, *p* < 0.05).

Subsequent experiments conducted after 12 h of OFFE treatment revealed that the ROS levels were significantly suppressed in all OFFE-treated and BAC groups compared with those in the control group, especially in the 0.6 mg/mL OFFE-treated group, which exhibited a 19% decrease in ROS fluorescence intensity (Figure 4C, *p* < 0.001). This suggests that OFFE treatment could effectively inhibit ROS production in 3T3-L1 cells. After 24 h of OFFE treatment, ROS fluorescence intensity of the 0.2 mg/mL OFFE-treated group increased by 45% (Figure 4D, *p* < 0.05), whereas the remaining groups exhibited diminished ROS fluorescence intensities compared to the control.

In summary, intracellular ROS levels measured at 4 time points revealed that OFFE induced a transient increase in intracellular ROS levels for a brief duration (<4 h), followed by significant inhibition of ROS accumulation in mature adipocytes after 12 h. These observations offer valuable insights into the modulation of ROS production and mitochondrial function in adipocytes.

### 2.5. OFFE Enhanced Mitochondrial Biogenesis and Induced Browning Marker Expression in Adipocytes

**Figure 5:**
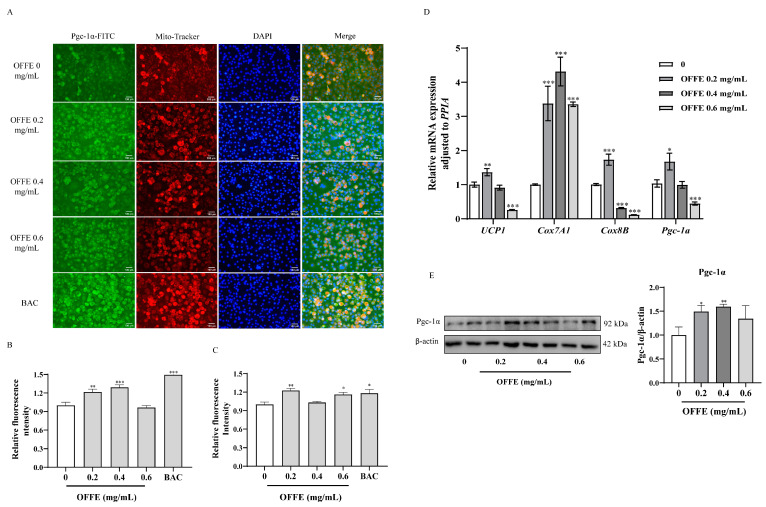
Effect of OFFE on mitochondrial biogenesis and adipocyte browning in 3T3-L1 adipocytes. Representative immunofluorescence images of 3T3-L1 adipocytes. (**A**) The distribution of adipocytes following OFFE treatment is shown (200× magnification, scale bar = 100 μm). Semi-quantitative immunofluorescence analysis of mitochondrial biogenesis (**B**) and Pgc-1α (**C**). (**D**) *UCP1*, *Cox7A1*, *Cox8B*, and *Pgc-1α* mRNA expression. (**E**) Pgc-1α protein levels in mature adipocytes treated with OFFE during differentiation. The values are expressed as the mean ± SEM of three independent experiments. * *p* < 0.05, ** *p* < 0.01, *** *p* < 0.001, compared with the control group.

Brown adipocytes contain a high number of mitochondria and play a crucial role in adipose tissue metabolism by promoting fat-burning and thermogenesis. Mitochondrial biogenesis is enhanced during white adipocyte browning or beiging. Therefore, mitochondrial biogenesis was assessed using selective fluorescence staining with MitoTracker Red CMXRos. The control group exhibited lower mitochondrial fluorescence intensity, which significantly increased following OFFE treatment. In the 0.4 mg/mL–treated group, mitochondrial morphology closely resembled that of differentiated cells in the BAC group (positive control) (Figure 5A). Furthermore, fluorescence intensity analysis demonstrated that OFFE treatment substantially enhanced mitochondrial biogenesis, especially in the 0.2 and 0.4 mg/mL treatment groups, with increases of 22% (*p* < 0.01) and 29% (*p* < 0.001), respectively, compared with that in the control group (Figure 5B), which was almost similar to the levels observed in the positive control group. These data indicate that OFFE treatment at low concentrations significantly enhanced mitochondrial biogenesis, facilitating white adipocyte browning/beiging.

PGC-1α plays a crucial role in the thermogenesis of brown adipose tissues. Immunofluorescence staining of OFFE-treated adipocytes showed that PGC-1α expression was dramatically increased (Figure 5A). Semi-quantitative analysis of the immunofluorescence intensity further validated our findings. Treatment with 0.2 mg/mL of OFFE resulted in a significantly higher immunofluorescence intensity of PGC-1α (23%) (Figure 5C, *p* < 0.01) than in the control group. This increase was 4% higher than that in cells differentiated using the beige adipocyte differentiation medium. Moreover, in the 0.4 and 0.6 mg/mL treatment groups, fluorescence intensity was significantly increased by 3% and 16% (Figure 5C, *p* < 0.05) compared to that in the control group.

Next, the expression of browning-specific marker genes *UCP1*, *Cox7A1*, and *Cox8B* was investigated. Compared to the control group, 0.2 mg/mL of OFFE significantly upregulated the mRNA expression of *UCP1* by 37% (Figure 5D, *p* < 0.01). OFFE significantly upregulated *Cox7A1* by 237%, 331%, and 235% at concentrations of 0.2, 0.4, and 0.6 mg/mL, respectively (Figure 5D, *p* < 0.001). In addition, the low concentration (0.2 mg/mL) treatment group exhibited a significant upregulation of *Cox8B* mRNA expression by 72% compared to that in the control group, whereas the medium and high concentrations significantly reversed these effects (Figure 5D, *p* < 0.001).

We also assessed the mRNA and protein expression of Pgc-1α using qRT-PCR and western blotting. As shown in Figure 5D, *Pgc-1α* mRNA expression was elevated by 62% (*p* < 0.05) upon OFFE treatment at 0.2 mg/mL compared to that in the control group. In addition, treatment with 0.2, 0.4, and 0.6 mg/mL of OFFE upregulated Pgc-1α levels by 49% (Figure 5E, *p* < 0.05), 60% (Figure 5E, *p* < 0.01), and 34% (Figure 5E), respectively, compared with that in the control group.

In summary, OFFE significantly enhanced mitochondrial biogenesis and elevated browning genes expression, thereby reducing white adipocyte production.

### 2.6. OFFE Inhibits Adipogenesis in 3T3-L1 Adipocytes by Activating AMPK

**Figure 6:**
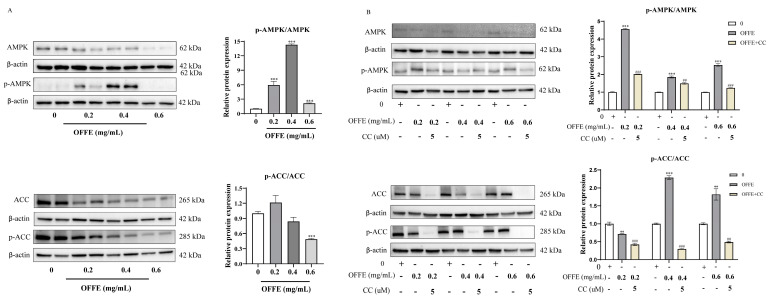
OFFE inhibited adipogenesis in 3T3-L1 adipocytes by activating AMPK. (**A**) Activation of AMPK and ACC phosphorylation in differentiated 3T3-L1 cells treated with different concentrations of OFFE. (**B**) Co−treatment with AMPK inhibitor Compound C (CC) for 24 h reversed the OFFE-induced upregulation of AMPK and ACC phosphorylation. The values are expressed as the mean ± SEM of three independent experiments. ** *p* < 0.01, *** *p* < 0.001, compared with the control group, ## *p* < 0.01, ### *p* < 0.001, i.e., OFFE combined with CC treatment group compared with the OFFE treatment group (**B**).

Western blotting indicated a significant upregulation of AMPK phosphorylation following OFFE treatment. The ratio of phosphorylated AMPK to total AMPK increased by 495%, 1331%, and 113% after treatment at concentrations of 0.2, 0.4, and 0.6 mg/mL, respectively (Figure 6A, *p* < 0.001). The ratio of phosphorylated to total acetyl-CoA carboxylase (ACC) increased by 21%, although this difference was not statistically significant (Figure 6A).

To investigate whether OFFE inhibited adipogenesis in 3T3-L1 adipocytes via the AMPK pathway, the phosphorylation status of AMPK and ACC was assessed using AMPK inhibitor compound C (CC). Co-treatment with CC and OFFE reversed the OFFE-induced AMPK and ACC phosphorylation. Co-treatment with various concentrations of OFFE and CC reduced AMPK phosphorylation levels by 55%, 22%, and 50% (Figure 6B, *p* < 0.001). These data indicate that OFFE attenuated adipocyte differentiation through AMPK pathway engagement.

## 3. Discussion

Adipocyte hypertrophy and hyperplasia are quintessential indicators of obesity’s progression, with adults typically having approximately 30 billion white adipocytes, each of which can store excess triglycerides. Adipocyte differentiation is critical to the progression of obesity, as an altered number or size of fully differentiated adipocytes result in excessive fat accumulation [34]. Overexpansion of the WAT disrupts the white-to-brown adipocyte ratio, prompting interest in brown adipocyte activation or the induction of white adipocyte browning to enhance energy catabolism and combat obesity [36,37]. Plant extracts rich in flavonoids show promise in promoting white adipocyte browning, boosting energy expenditure, countering high-fat diet-induced obesity, and improving metabolic function [38,39].

A growing focus on the health benefits of adipocyte browning has driven research into relevant pharmaceuticals and natural compounds [40,41]. Despite the approval of synthetic anti-obesity drugs, their side effects remain significant [42,43]. Edible Chinese medicines have attracted considerable attention owing to their high efficiency, low toxicity, and multi-target bioactive components [44,45].

Over 5000 flavonoids have been identified in plants, recognized as a significant group of natural substances with substantial bioactive potential [46]. Flavonoids have been shown to inhibit preadipocyte differentiation and reduce lipid accumulation in 3T3-L1 adipocytes [27]. Isorhamnetin interventions in high-fat diet (HFD)-fed mice can attenuate weight gain and improve lipid accumulation by decreasing the expression of lipogenic genes in white adipose tissue [47]. Additionally, wogonin (5,7-dihydroxy-8-methoxyflavone) from Scutellaria baicalensis has been found to reduce body weight and ameliorate nonalcoholic fatty liver disease in obese mice [48]. In addition, 5,7-Dimethoxyflavones have also been shown to reduce HFD-induced weight gain and decrease the protein expression of transcription factors involved in adipogenesis in adipose tissues [49].

The anti-obesity potential of flavonoids is highlighted by appetite control, a reduction in food intake and intestinal fat absorption, modulation of metabolic processes (i.e., adipogenesis, lipolysis, and β-oxidation), induction of non-shivering thermogenesis, stimulation of energy expenditure, and modulation of the intestinal flora [50]. Several flavonoid components found in extract of *O. fragrans* were reported to have anti-diabetic and anti-obesity efficacy. Phenylpropyl triterpenoids 2–4, isolated from the leaves of *O. fragrans*, have demonstrated both preventive and therapeutic effects against inflammatory bowel disease [51]. Also, 9,12-Octadecadienoic acid, 4-(2,6,6-trimethyl-1-cyclohexenyl)-3-buten-2-one, and their analogs exhibit obvious inhibitory activity against α-glucosidase, suggesting a potential antidiabetic effect [26,52]. Acteoside, the major bioactive compound in *O. fragrans* flowers, ameliorates intestinal inflammation, oxidative stress, and nuclear factor-κB (NF-κB) activation in mice with colitis [53]. Herein, we report for the first time that flavonoid extracts from *O. fragrans* inhibit adipogenesis via the AMPK pathway, scavenge ROS, stimulate mitochondrial biogenesis, and promote the browning/beiging of white adipocytes.

AMPK, a cellular energy sensor regulating lipid metabolism, is a promising therapeutic target for metabolic disorders. Its activity, modulated by changes in the AMP/ATP ratio during cellular stress, inhibits adipogenesis by phosphorylating and inactivating ACC, reducing serum-free fatty acids and fat deposition. AMPK also modulates SREBP-1C, a key lipid metabolism regulator. Many bioactive compounds inhibit adipogenesis via the AMPK-ACC pathway [54,55,56,57]. This study assessed AMPK expression and phosphorylation after OFFE treatment, finding that OFFE suppressed C/EBPα, FASN, and SREBP-1C in 3T3-L1 adipocytes and upregulated AMPK phosphorylation. Compound C and OFFE together reversed AMPK phosphorylation, indicating OFFE’s lipid-lowering effect is AMPK-mediated, consistent with Bu et al. [54].

Recent studies have indicated that the generation of ROS is linked to the pathogenesis and progression of numerous diseases, notably obesity and metabolic syndrome [58,59]. Excessive accumulation of lipids in adipocytes results in increased levels of intracellular ROS, which can lead to mitochondrial dysfunction by damaging proteins, membrane lipids, and nucleic acids [60]. It has been reported that both concentrated extract of *Prunus mume* fruit and 3-hydroxymorphinan can inhibit ROS generation in 3T3-L1 adipocytes to improve mitochondrial function [38,61]. Similarly, the results of the present study indicated that OFFE reduced intracellular ROS levels in a dose- and time-dependent manner, shielding mitochondria from oxidative-stress-induced harm and thereby preserving mitochondrial function.

Furthermore, OFFE treatment led to a significant upregulation of the brown/beige adipocyte marker Pgc-1α in mature 3T3-L1 adipocytes (Figure 5D). This was accompanied by a notable increase in the expression of key browning genes, including *UCP1*, *Cox7A1*, and *Cox8B* (Figure 5D). Additionally, Pgc-1α protein levels exhibited a significant increase, as confirmed via immunofluorescence staining and western blotting (Figure 5A,E). Pgc-1α, a transcriptional co-activator, orchestrates various biological activities associated with energy metabolism. It plays a pivotal role in regulating the expression of UCP1 and thermogenesis in brown adipose tissues. Moreover, it controls mitochondrial biogenesis and oxidative metabolism in diverse cell types [62]. The cytochrome C oxidase subunit isoform Cox7A1, a component of the cytochrome C oxidase complex, is found in high amounts in skeletal muscle and myocardium. It modulates enzyme activities that are characterized by a high oxidative capacity in these tissues. The associated pathways include AMPK signaling, respiratory electron transfer, ATP synthesis via chemo-osmotic coupling, and heat production facilitated by uncoupling [63,64]. Although the increase in UCP1 protein levels of OFFE-treated 3T3-L1 adipocytes was not confirmed via western blotting, this may be explained by the fact that beige adipocytes resemble white adipocytes with very low UCP1 basal expression. However, similar to classical brown fat cells, beige adipocytes exhibit a robust response to cyclic AMP stimulation, resulting in elevated UCP1 expression and increased respiratory activity [65].

Other pathways, such as PKA, PLC-IP3, and MAPK, might be involved in the lipid accumulation and adipocyte browning induced by OFFE, which requires further study. In addition, the specific bioactive components of OFFE responsible for the observed effects remain to be determined.

## 4. Materials and Methods

### 4.1. Extraction and Composition Analysis of OFFE

The preparation and compositional analysis of OFFE were performed at Dr. Tingting Li’s lab in the College of Light Industry and Food Science, Nanjing Forestry University, China. The crude flavonoids of *O. fragrans* were extracted by 52% ethanol with ultrasound (200 W) assistance for 60 min at 50 °C, with the liquid-solid ratio of 17:1 (mL:g). The monomeric components were then identified by ultra-performance liquid chromatography-tandem mass spectrometry (UPLC-MS/MS) [66]. The main composition of OFFE is presented in Table 1.

### 4.2. Chemicals and Reagents

Dulbecco’s Modified Eagle Medium (DMEM) high-glucose (4.5 g/L) medium, DMEM low-glucose (1 g/L) medium, Dulbecco’s phosphate-buffered saline solution (DPBS), fetal bovine serum (FBS), penicillin–streptomycin solution, and 0.25% trypsin solution were provided by Gibco (Rockville, MD, USA). Primary antibodies specific for FASN, C/EBP α, GLUT-4, CPT1α, AMPK, ACC, and p-ACC were obtained from Cell Signaling Technology Danvers, MA, USA). Anti-SREBP-1c and anti-Pgc-α were purchased from Abcam (Cambridge, UK). Anti-p-AMPK was obtained from Wanleibio (Shenyang, China). Anti-β-Actin was obtained from Trans-Gen Biotech (Beijing, China). Goat anti-mouse and goat anti-rabbit IgG secondary antibodies were purchased from Abmart (Shanghai, China). The 4,6-diamidino-2-phenylindole (DAPI), ROS assay kit, and the MitoTracker Red CMXRos Assay Kit were purchased from Beyotime Biotechnology (Shanghai, China). Compound C was purchased from Selleck Chemicals (Houston, TX, USA). The OFFE was provided by Dr. Tingting Li’s team from the School of Light Industry and Food, Nanjing Forestry University, China [66].

### 4.3. Cell Culture, Differentiation, and OFFE Treatment

The 3T3-L1 cell line (SCSP-5038) was acquired from Shanghai’s National Collection of Authenticated Cell Cultures, affiliated with the China Academy of Sciences (Shanghai, China). Induction of maturation in 3T3-L1 pre-adipocytes was performed with an established laboratory protocol [38]. Unless otherwise specified, varying concentrations of OFFE were incorporated into differentiation medium II throughout the differentiation process.

To stimulate beiging/browning, 3T3-L1 pre-adipocytes underwent differentiation in beige differentiation medium I (0.5 mM IBMX, 0.5 μM DEX, 4 μg/mL insulin, 50 nM T3, and 1 μM Rosi) for a duration of 2 days. This was then switched to beige differentiation medium II (4 μg/mL Insulin, 50 nM T3), and the described process was repeated at two-day intervals until reaching day eight. Beiging/browning differentiation medium-induced adipocytes were used as a positive control [38]. In relevant experiments, adipocytes undergoing beige differentiation (BAC) were used as positive controls.

### 4.4. Cytotoxicity Assay

Cultivation of 3T3-L1 pre-adipocytes proceeded in 96-well plates, achieving 80% confluence post-overnight incubation. The cells were cultured in DMEM with 1 mg/mL glucose, supplemented by 10% FBS and 1% Pen-Strep, introducing varying OFFE levels (0–0.5 mg/mL) over a 48-h period. Similarly, pre-adipocytes were differentiated in 96-well plates followed the protocol described above and then treated with varying concentrations of OFFE for 48 h. Following treatment, each well received 50 µL MTT solution at 1 mg/mL concentration. The mixture was incubated for 4 h at 37 °C with 5% CO_2_. The cells were washed, added with 200 μL of dimethyl sulfoxide (DMSO) and agitated at 800 rpm for 10 min to solubilize formazan, followed by absorbance assessment at 550 nm (Synergy 2, BioTek, Winooski, VT, USA). Calculate cell survival using the following formula: cell viability = (readings per sample − zeroed average)/(control average − zeroed average) × 100% [67].

### 4.5. Oil Red O Staining

Lipid droplets in mature adipocytes were assessed with or without OFFE (0, 0.2, 0.4 and 0.6 mg/mL) treatment during the differentiation process, utilizing Oil Red O (ORO) staining. Adipogenic differentiation of 3T3-L1 cells was conducted in 24-well culture plates. On day 8 post-differentiation, cells underwent dual washes in 1 × DPBS, a 30-min 10% formaldehyde fixation, followed by another 1 × DPBS wash. Staining was performed by adding 200 µL of ORO working solution (0.6 mg/mL) to each well and incubated under ambient conditions for one hour. Post-stain excess was eliminated via sterile aqueous lavage. Photographic documentation was accomplished via an inverted fluorescence microscopy technique (Ti2U, Nikon, Tokyo, Japan). Following the initial procedure, each well was supplemented with 100 µL of isopropanol. The mixtures were shaken at 500 rpm and 37 °C for 5 min to fully dissolve the ORO in isopropanol. Exactly 50 µL of ORO solution was transferred from each well to a 96-well plate, followed by measuring absorbance at 510 nm to quantify the lipid droplet content in 3T3-L1 adipocytes.

### 4.6. Extracellular Triglyceride Assay

To examine the extracellular triglyceride content of mature adipocytes treated with OFFE (0.2, 0.4, and 0.6 mg/mL during the differentiation process), on day 7 of differentiation, adipocytes underwent dual DPBS washes and received a 48-h (D7-D8) OFFE treatment. The medium was harvested by centrifuging at 1000 rpm for 10 min. Precisely 5 µL supernatant was aliquoted into microplate wells. Triglyceride levels were measured with the TG assay kit (Nanjing Jiancheng Bioengineering Institute, Nanjing, China). Following the manufacturer’s guidelines, the working solution was prepared and combined with the supernatant. After a 10-min reaction at 37 °C, the absorbance was measured at 500 nm using a BioTek Multi-Mode Microplate Reader (Synergy2, Vineland, NJ, USA). The triglyceride content was calculated according to the kit (Jiancheng Nanjing, Jiangsu, China) instructions using the following formula: triglyceride content (mmol/gprot) = (A_sample_ − A_blank_)/(A_standard_ − A_blank_) × C_standard_/Cpr (C_standard_: standard concentration; Cpr: protein concentration in the tissue sample homogenate to be tested, gprot/L).

### 4.7. Intracellular ROS Staining

Intracellular ROS production in mature adipocytes after OFFE treatment were assessed at various time points using an ROS assay kit (Beyotime, Shanghai, China). The procedure was followed as previously described [68]. The 3T3-L1 pre-adipocytes were differentiated in 12-well culture plates. The mature adipocytes were treated with OFFE at concentrations of 0, 0.2, 0.4, and 0.6 mg/mL. The cells were kept in a 37 °C, 5% CO_2_ incubator for 0.5, 4, 12, and 24 h. They were then collected using a 0.25% trypsin-EDTA solution. The cells were resuspended and stained in a 10 μM DCFH-DA solution at 37 °C for 20 min in the dark, and then washed several times in serum-free media. The ROS fluorescence quantification ensued via a BioTek Reader (Synergy2, Vineland, NJ, USA) at 480/525 nm excitation/emission, normalized against protein content.

### 4.8. Mitochondrial Biogenesis

Mitochondrial biogenesis in 3T3-L1 adipocytes was assessed with OFFE exposure from differentiation days 3–8. MitoTracker Red CMXRos dye (1 mM) was diluted in differentiation medium III to obtain a 100 nM working solution. The cells were then stained at 37 °C, 5% CO_2_ in the dark. The stained mitochondria were imaged using a fluorescence microscope (Ti2U; Nikon, Japan).

### 4.9. Immunofluorescence

Immunofluorescence staining followed established protocols [35]. Briefly, OFFE at 0, 0.2, 0.4, and 0.6 mg/mL was incorporated into the differentiation medium from days 3 to 8. The cells were permeabilized for 10 min using 0.1% Triton X-100 (dissolved in 1 × DPBS), followed by three rinses with 1 × DPBS. Subsequently, 5% Bovine Serum Albumin (BSA) in DPBS was added and incubated for 1 h. The anti-Pgc-1α primary antibody (1:300 dilution) in 1% BSA was added and incubated overnight at 4 °C. Goat Anti-Mouse IgG (H&L)-Alexa Fluor 488 (1:200 dilution in 1% BSA) was then incubated for 1 h at 4 °C in the dark (covered with tin foil). After three washes with 1 × DPBS, DAPI staining solution (Beyotime, Shanghai, China) was added and stained for 5 min at 25 °C, followed by four washes with 1 × DPBS. Finally, a small amount of 1 × DPBS was added, and the cells were observed under an inverted fluorescence microscope.

### 4.10. Quantitative Real-Time PCR Analysis

Total RNA was extracted using a SteadyPure Universal RNA Extraction Kit (AG, Changsha, China), and the RNA concentration was assessed using a Nanodrop ND-1000 microspectrophotometer (NanoDrop Technologies, Wilmington, DE, USA). cDNA was synthesized via reverse transcription following the protocol of Evo M-MLV Reverse Transcription Reagent Premix (for qPCR) Kit (AG, Nanjing, China). The reverse transcription of 500 ng total RNA was conducted in a 20 μL solution at 37 °C for 45 min, with subsequent enzyme deactivation at 85 °C for 1 minute. The gene expression levels were assessed using an SYBR Green Pro Taq HS Premixed qPCR Kit (AG, Nanjing, China) on a StepOnePlus Real-Time PCR System (Applied Biosystems Inc., Foster City, CA, USA). Data were analyzed via relative quantification (2^−ΔΔCT^), with the change in target gene expression calculated using peptidyl prolyl isomerase A (*PPIA*) as an internal reference. Table 2 presents the primers used for this study.

### 4.11. Immunoblotting

Protein isolation and immunoblot analyses proceeded in accordance with established protocols [65]. Total cellular proteins (30 µg) were separated via 10% SDS-PAGE, followed by their immobilization on PVDF membranes (Bio-Rad, Hercules, CA, USA). The membranes were then blocked with 5% non-fat milk in TBST buffer for 1 h. Specific primary antibodies against C/EDPα (1:1000, abcam), SREBP-1C (1:1000, abcam), FASN (1:1000, CST), Pgc-1α (1:1000, abcam), AMPK (1:1000, CST), p-AMPK (1:1000, CST), ACC (1:1000, CST), and p-ACC (1:1000, CST) were incubated with the membrane at 4 °C overnight. After three times’ TBST wash, the membrane was incubated with a secondary antibody (Abmart, Nanjing, China) for 1 h at room temperature. The immunoreactive bands were analyzed by chemiluminescence with the ClarityTM West ECL substrate (Bio-Rad, Hercules, CA, USA). The signal was quantified via densitometry using ImageJ software (ImageJ 1.53t) (National Institutes of Health, NIH, Bethesda, MD, USA).

### 4.12. Statistical Analysis

Data were analyzed using GraphPad Prism 9.5.1 software (GraphPad Software Inc., La Jolla, CA, USA) and are presented as the mean ± SEM. A multi-group comparison was conducted using one-way analysis of variance with a Student–Newman post-hoc test. All experiments were performed in three biological replicates unless otherwise stated. Statistical significance was set at * *p* < 0.05, ** *p* < 0.01, and *** *p* < 0.001,

## 5. Conclusions

In this study, we investigated the inhibitory effect of OFFE on adipogenesis and related expression in 3T3-L1 adipocytes. Firstly, OFFE exerts its effect by inhibiting the accumulation of lipid droplets during differentiation, stimulating the release of extracellular triglyceride content. Secondly, OFFE inhibits the mRNA and protein expression levels of transcription factors (C/EBPα and SREBP-1C) and lipid synthase (FASN), which are key genes in the process of fat synthesis and accumulation, thus exerting an inhibitory effect on adipogenesis. Interestingly, this inhibitory effect is achieved by upregulating AMPK and ACC. Moreover, OFFE stimulated mitochondrial biogenesis and promoted the upregulation of several BAT biomarkers, mitigating the release of ROS and enhancing white adipocyte beiging/browning. Collectively, our findings suggest that OFFE has the potential to inhibit adipogenesis and promote white adipocyte beiging/browning. Therefore, the study proposes OFFE as a promising candidate for further experiments in mice and humans as an alternative anti-obesity therapy.

## Figures and Tables

**Table 1:** Main chemical constituents of OFFE determined via UPLC-MS/MS.

Serial Number	Chemical Formula	Compound	Relative Content
1	C_17_H_16_O_5_	4-Hydroxy-5,7-dimethoxyflavanone	12.13
2	C_27_H_30_O_17_	Baimaside	3.59
3	C_27_H_30_O_17_	6-Hydroxykaempferol-3,6-O-diglucoside	3.88
4	C_27_H_30_O_16_	Rutinum	2.52
5	C_27_H_30_O_16_	Quercetin 3-O-glucoside-7-O-rhamnoside	2.73
6	C_27_H_30_O_16_	Quercetin 3-O-neohesperidoside	2.43
7	C_27_H_30_O_16_	Quercetin 3-O-robinobioside	1.83
8	C_21_H_20_O_12_	6-Hydroxykaempferol 3-O-beta-D-glucoside	0.33
9	C_27_H_30_O_14_	Rhoifolin	1.81
10	C_27_H_30_O_14_	Apigenin-7-rutinoside	2.4

**Table 2:** Primers used for qRT-PCR.

Genes	Primer Sequence	Accession No.
*PPIA*	Forward: 5′-GAGCTGTTTGCAGACAAAGTTC-3′ 666 Reverse: 5′-CCCTGGCACATGAATCCTGG-3′	NM-00897.2
*C/EBPα*	Forward: 5′-GCCCCCGTGAGAAAAATGAAG-3′ 666 Reverse: 5′-GAGGTGCGAAAAGCAAGGGA-3′	NM_001308354.1
*FASN*	Forward: 5′-ATTCGGTGTATCCTGCTGTC-3′ 666 Reverse: 5′-GCTTGTCCTGCTCTAACTGG-3′	NM_007988.3
*SREBP-1c*	Forward: 5′-TGGACTACTAGTGTTGGCCTGCTT-3′ 666 Reverse: 5′-ATCCAGGTCAGCTTGTTTGCGATG-3′	NM_001278601
*GLUT-4*	Forward: 5′-AGCCTCTGATCATCGCAGTG-3′ 666 Reverse: 5′-ACCGAGACCAACGTGAAGAC-3′	NM_009204
*Pgc-1α*	Forward: 5′-CCCTGCCATTGTTAAGACC-3′ 666 Reverse: 5′-TGCTGCTGTTCCTGTTTTC-3′	XM_006503779.3
*Cox7A1*	Forward: 5′-AGCTCTTCCAGGCCGACAAT-3′ 666 Reverse: 5′-GAGTCAGCGTCATGGTCAGT-3′	NM_009944.3
*Cox8B*	Forward: 5′-AGCCAAAACTCCCACTTCC-3′ 666 Reverse: 5′-TCTCAGGGATGTGCAACTTC-3′	NM_007751.3
*UCP1*	Forward: 5′-ACTGCCACACCTCCAGTCATT-3′ 666 Reverse: 5′-CTTTGCCTCACTCAGGATTGG-3′	XM_021170845.1

## Data Availability

The original contributions presented in the study are included in the article, further inquiries can be directed to the corresponding author.
